# Structural and functional characterization of suramin-bound MjTX-I from *Bothrops moojeni* suggests a particular myotoxic mechanism

**DOI:** 10.1038/s41598-018-28584-7

**Published:** 2018-07-09

**Authors:** Guilherme H. M. Salvador, Thiago R. Dreyer, Antoniel A. S. Gomes, Walter L. G. Cavalcante, Juliana I. dos Santos, César A. Gandin, Mário de Oliveira Neto, Márcia Gallacci, Marcos R. M. Fontes

**Affiliations:** 1 Universidade Estadual Paulista (UNESP), Instituto de Biociências, Dep. de Física e Biofísica, Botucatu, SP Brazil; 2 Universidade Federal de Minas Gerais (UFMG), Instituto de Ciências Biológicas, Dep. de Farmacologia, Belo Horizonte, MG Brazil; 3 Universidade Estadual Paulista (UNESP), Instituto de Biociências, Dep. de Farmacologia, Botucatu, SP Brazil

## Abstract

Local myonecrosis is the main event resulting from snakebite envenomation by the *Bothrops* genus and, frequently, it is not efficiently neutralized by antivenom administration. Proteases, phospholipases A_2_ (PLA_2_) and PLA_2_-like toxins are found in venom related to muscle damage. Functional sites responsible for PLA_2_-like toxins activity have been proposed recently; they consist of a membrane docking-site and a membrane rupture-site. Herein, a combination of functional, biophysical and crystallographic techniques was used to characterize the interaction between suramin and MjTX-I (a PLA_2_-like toxin from *Bothrops moojeni* venom). Functional *in vitro* neuromuscular assays were performed to study the biological effects of the protein-ligand interaction, demonstrating that suramin neutralizes the myotoxic effect of MjTX-I. Calorimetric assays showed two different binding events: *(i)* inhibitor-protein interactions and *(ii)* toxin oligomerization processes. These hypotheses were also corroborated with dynamic light and small angle X-ray scattering assays. The crystal structure of the MjTX-I/suramin showed a totally different interaction mode compared to other PLA_2_-like/suramin complexes. Thus, we suggested a novel myotoxic mechanism for MjTX-I that may be inhibited by suramin. These results can further contribute to the search for inhibitors that will efficiently counteract local myonecrosis in order to be used as an adjuvant of conventional serum therapy.

## Introduction

Ophidian accidents represent an important public health problem in rural areas of Asia, Africa and Latin America, where the number of deaths caused by snakebites are higher than other neglected tropical diseases, such as dengue haemorrhagic fever, cholera, leishmaniasis, schistosomiasis and Chagas disease. These facts lead the World Health Organization^1–3^ to classify snakebites as a neglected disease, increasing the interest of the scientific community to study the compounds of these venoms and their eventual neutralization by specific inhibitors.

In Latin America, the *Bothrops* genus is responsible for approximately 85% of all ophidian accidents^2,4^, with myonecrosis a major event of this envenoming, which is mainly caused by the association of two proteins classes: metalloproteinases and phospholipases A_2_^5–12^. Two main phospholipase A_2_ (PLA_2_) subclasses are often found in bothropic venoms: catalytic PLA_2_s and the myotoxic PLA_2_-like toxins. PLA_2_-like proteins are catalytically inactive proteins due to some natural amino acid substitutions, including Asp49Lys and Tyr28Asn, leading to Ca^+^^2^ coordination inability^13,14^.

The search for new inhibitors for PLA_2_-like proteins has been intensified in recent years^15–22^. Some of these known inhibitors are derived from medicinal plants (used in folk medicine, since some communities in developing countries do not have ready access to serum therapy)^23,24^, and others are synthetic compounds such as suramin (8,8′-[carbonylbis [imino-3,1-phenylenecarbonylimino (4-methyl-3,1-phenylene) carbonylimino]] bis-1,3,5-naphtalenetrisulfonic acid hexasodium salt). This synthetic highly charged polysulfonated compound has been clinically used in the treatment of African trypanosomiasis and onchocerciasis^25,26^. Regarding its anti-ophidic activity, it has been shown that suramin is able to inhibit neuromuscular blockage induced by pre-synaptic neurotoxins, such as crotoxin and *β-*bungarotoxin^27,28^, and to prevent muscle necrosis promoted by PLA_2_-like proteins from *Bothrops* snake venom^17,22,29^. It is expected that, in the near future, some of these studied inhibitors (or their modified versions) may be approved as compounds to be used as complements to the conventional serum therapy^20^.

Important regions that can be essential for myotoxic activity in bothropic PLA_2_-like proteins were recently observed^14,30–33^. Some of these regions comprise the myotoxic site (Lys20, Lys115 and Arg118) — subsequently called the membrane docking site (MDoS) — and the membrane disruption site (MDiS) composed of the conserved residues Leu121 and Phe125^33^. Despite these hypotheses being applied to the majority of bothropic PLA_2_s-like toxins, MjTX-I is an exception^34^ because its sequence does not present all the MDoS residues, and its native crystal structure exhibits a different oligomeric configuration. These factors were noted as possible factors for MjTX-I reduced myotoxicity when compared to other PLA_2_-like toxins^34^.

Recently, functional, calorimetric and structural studies with the MjTX-II/suramin complex were performed and showed the inhibition of MjTX-II myotoxic activity by suramin was due to two different mechanisms: *(i)* direct blockage of the MDoS and MDiS, preventing the toxin/membrane interaction and disruption; and *(ii)* formation of an oligomeric complex, resulting in a tetrameric configuration for which both MDoS and MDiS becomes buried (physically inaccessible), thus avoiding any possibility of toxin-membrane interaction or disruption^22^.

In this work, we studied the binding behaviour between MjTX-I, a myotoxic PLA_2_s-like isolated from *Bothrops moojeni* venom, and the suramin molecule using a broad combination of techniques, including myographic assays, molecular dynamic simulations, dynamic light scattering (DLS), small angle X-ray scattering (SAXS), isothermal titration calorimetry (ITC) and X-ray crystallography. In the functional studies, after adding suramin to the protein solution, the MjTX-I myotoxic activity was considerably reduced in muscle preparations. Data from ITC experiments showed that interaction between suramin molecules and MjTX-I occurred, inducing protein oligomerization, a process that was also corroborated by DLS and SAXS experiments. The crystal structure of the complex MjTX-I/suramin revealed remarkable differences when compared to the native MjTX-I structure, which were mainly due to the ligand interactions. Based on the data produced and comparisons with the literature, we propose a particular myotoxic mechanism for MjTX-I and its inhibition by suramin, thus providing insights that may be very useful in the search for new components to enhance serum therapy effectiveness, particularly for bothropic snake bites.

## Results

### Functional assays

**Figure 1:**
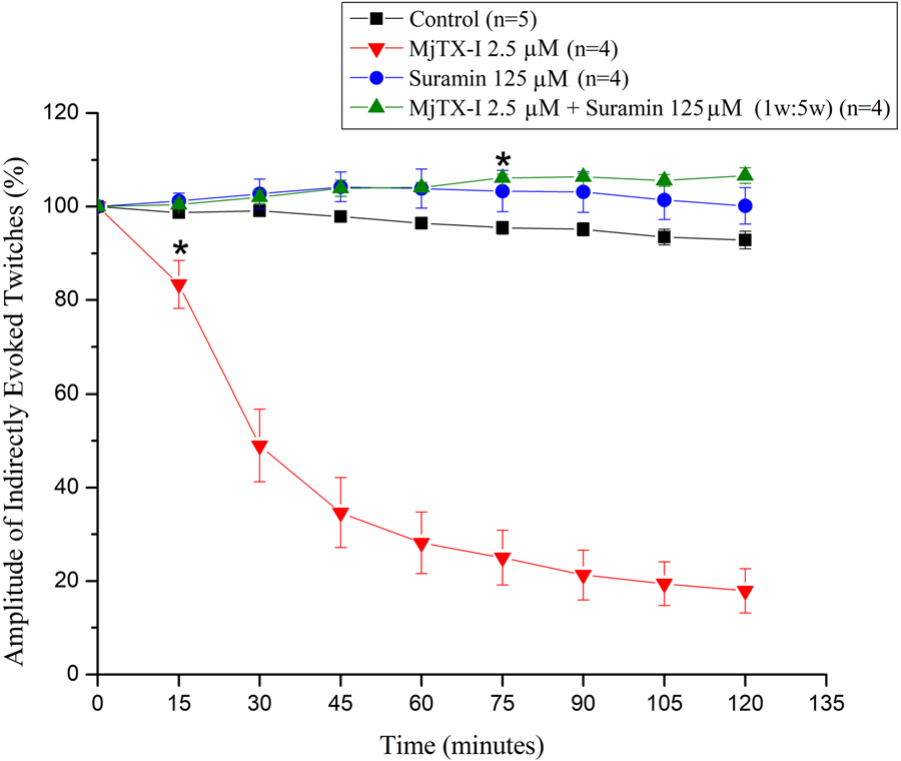
Effects of MjTX-I and the product of its pre-incubation with suramin on indirectly evoked twitches in mice phrenic diaphragm preparations. The ordinate represents the % of twitches relative to the initial amplitude. The abscissa indicates the time (minutes) after the addition of MjTX-I, suramin or the mixture of MjTX-I plus suramin to the organ bath. The data are grouped as means ± SEM (P < 0.05). *Indicates the point from which there was a significant difference compared with control.

MjTX-I (2.5 μM) induced a time-dependent blockade of indirectly evoked twitches in mouse neuromuscular preparations (Fig. 1). Twitch amplitudes were depressed in approximately 80% after 120 minutes of toxin contact with the preparation. The time required for 50% reduction of twitch amplitudes (t_1/2_) was 32.5 ± 5.6 minutes (n = 4). After pre-incubation with suramin (125 μM) for 15 minutes, MjTX-I did not depress the twitch amplitudes; instead, it induced a discrete but significant facilitation of twitches starting at 75 minutes of toxin contact. Alone, suramin did not alter muscle contractions compared to the controls.

### MjTX-I-suramin affinity assays

**Figure 2:**
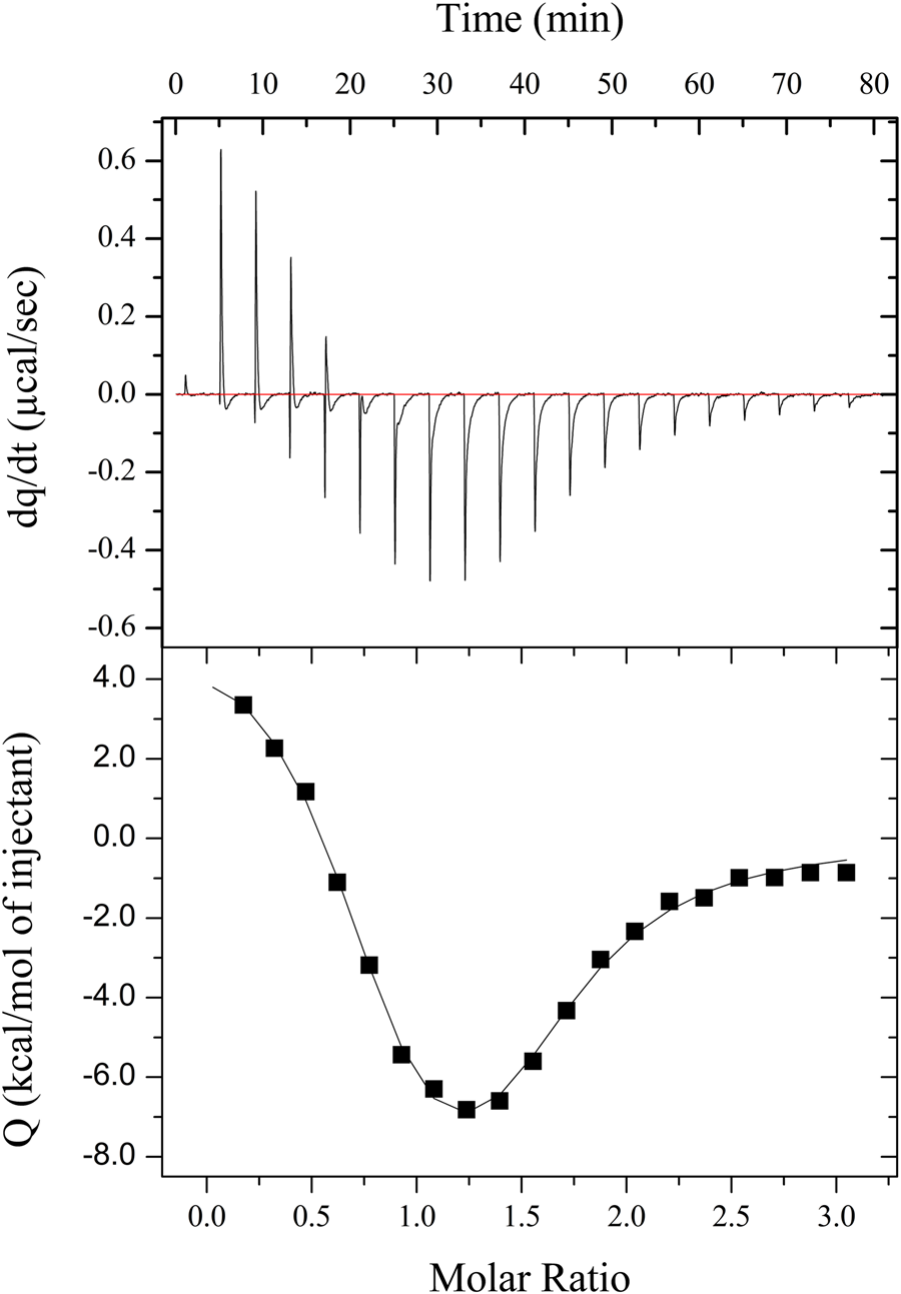
Isothermal titration calorimetry data for the reaction of MjTX-I and suramin. Upper panel presents the titration raw data for the interaction between MjTX-I (50 μM, reaction cell) and suramin (750 μM, pipette) at 25 °C in ammonium bicarbonate (50 mM, pH 8.0). Lower panel shows the binding isotherm (squares) and the general nonlinear regression model considering two binding events (solid line).

MjTX-I/suramin interactions were assessed with isothermal titration calorimetry (ITC) experiments. The titrations presented a biphasic behaviour which is characteristic of more than one binding event, with the enthalpic component varying from endo- to exothermic (Fig. 2a). The binding isotherm was adjusted through a nonlinear regression model considering two binding events, as previously described^22,35^, resulting in an interaction constant *α* (=${4\beta }_{2}/{\beta }_{1}^{2}$) and Hill coefficient [=2/(1+ *α*^−1/2^)] of 0.2 and 0.6, respectively, which indicates non-identical binding or identical binding events with negative cooperativity^36^. According to the crystallographic structure, suramin binds to both protomers of the MjTX-I homodimeric structure making different contacts in each protomer (Fig. 2b), so we considered the binding events as non-identical. For the sake of simplicity in the adjusted model, both events of suramin binding to monomers A or B of MjTX-I (MjTX-I/suramin, 1:1 molecular ratio) were considered as a general binding event, and the same was applied to the second event (MjTX-I/suramin, 2:1). Based on these assumptions, the specific dissociation constants and enthalpy changes were calculated and are summarised in Table 1.

**Table 1:** Thermodynamic-binding data of MjTX-I and suramin.

Specific dissociation and binding enthalpy changes	
k_d1_ (μM)	0.5 ± 0.2
ΔH_1_ (kcal/mol)	6.0 ± 0.9
k_d2_ (μM)	6.0 ± 1.0
ΔH_2_ (kcal/mol)	−14.8 ± 2.9

The estimated specific dissociation constants (k_d_) and enthalpy changes (ΔH) were determined for the binding of suramin (750 μM) to MjTX-I (50 μM) at 25 °C in ammonium bicarbonate buffer (50 mM, pH 8.0) by binding polynomials. Index 1 and 2 are related to the binding events of MjTX-I/suramin (1:1, molecular ratio) and MjTX-I/suramin (2:1), respectively.

Calorimetric data showed that suramin binds entropically in the submicromolar range to MjTX-I monomers, while the second binding event, related to dimer formation, presents 10-fold less affinity and is enthalpically driven (Table 1). Further considerations about enthalpy changes will not be made since suramin is a highly charged polysulfonated molecule, which could lead to proton exchange and ionization, making the binding enthalpy itself different from the observed enthalpy. However, this unusual binding pattern (i.e., non-sigmoidal binding isotherm) was not classified as an artefact since the behaviour was observed in all titrations that were performed and were repeated at least twice with each protein and the suramin dilution heat was constant in the blank titration.

### Quaternary structure in solution

Dynamic light scattering (DLS) experiments showed that native MjTX-I was predominantly monomeric when dissolved at 2.5 mg.mL^−1^ in 20 mM of ammonium bicarbonate (pH 8.5), with an unimodal molecular distribution (Pd = 13.1%) and a hydrodynamic radius (R_H_) of 2.0 nm. On the other hand, the DLS measurements performed in the same conditions, but with pre-incubation of the protein with suramin (1:10, molecular ratio), indicated molecular oligomerization. The results presented a unimodal molecular distribution (Pd = 11.3%) with a R_H_ of 3.4 nm, suggesting the oligomerization of MjTX-I in the presence of suramin.

**Figure 3:**
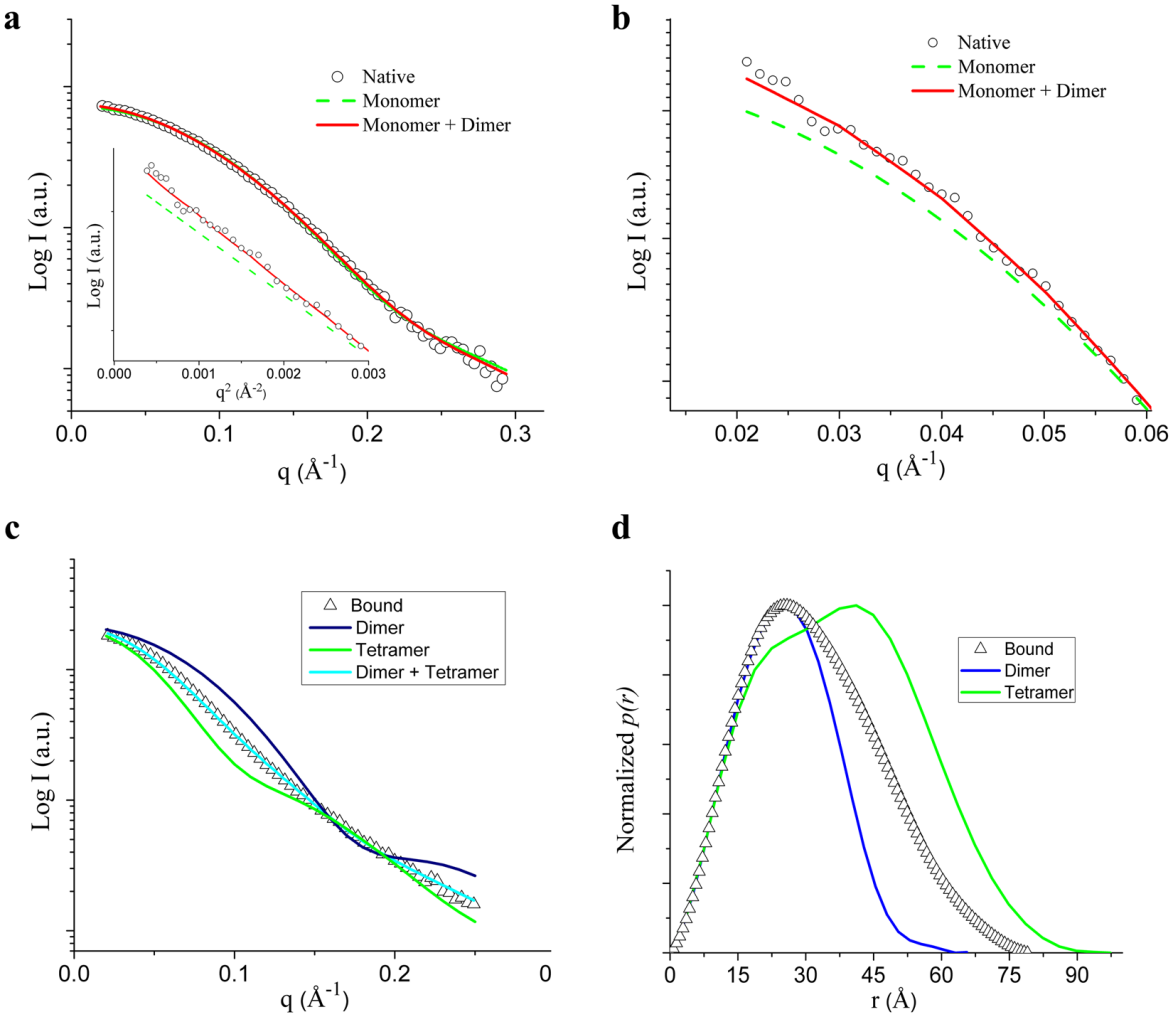
Fits to the experimental SAXS data. (**a**) Simulated SAXS from the monomeric crystallographic structure and from the monomer/dimer mixture evaluated from Oligomer. Insert - Guinier plot of both fits demonstrating the increase of the R_g_ parameter (slope). (**b**) Zoom in the small angle region, demonstrating the better adjustment proposing a small percentage of dimers in solutions. (**c**) Dimer and tetramer simulated SAXS profile and dimer/tetramer fit against the experimental bound data. (**d**) *P(r)* of the experimental bound SAXS data and from the simulated SAXS of the dimeric and tetrameric structures.

SAXS experiments were performed to obtain structural information of MjTX-I in solution in the absence (native) and the presence of suramin (complexed state). Structural parameters obtained from SAXS analyses suggested that the native MjTX-I behaves as a monomer in solution (Table 2); however, the simulated scattering from the monomeric crystallographic structure (generated from PDB id: 3T0R) fitted the data with a high value of χ^2^ (12.8) due to an upturn of the data points in the Guinier region (Fig. 3a,b), suggesting minor oligomerization. Since the SAXS profile is an average of all conformations of the scattering particles in solution, we used Oligomer software^37^ to describe our results from the mixture of monomers/dimers in solution. Therefore, the experimental profile was better described by a 92.9%/7.1% monomeric/dimeric configuration evidenced by the decrease in the χ^2^ value from 12.8 (monomer fit) to 3.18 (monomer + dimer fit) (Fig. 3a,b). The insert in Fig. 3a displays a Guinier plot for both fits, confirming an increase in the radius of gyration (Rg) parameter (Table 2), which better describes the experimental results. In the presence of suramin, the increase in the structural and molecular weight parameters due to conformational changes (oligomerization) suggested a conformation between a dimer and a tetramer, but the dimeric and tetrameric models alone did not fit the data. Thus, Oligomer software^37^ was used to describe the experimental results by a proposed dimer/tetramer mixture and indicated the presence of 48.9% dimers and 51.1% tetramers in solution, fitting the data with a χ^2^ of 1.8, showing that the presence of the inhibitor favours oligomerization (Fig. 3c). The pair-distance distribution function *p(r)* of the experimental bound SAXS data and the simulated SAXS of the dimeric and tetrameric structures are shown in Fig. 3d, demonstrating the almost 50%/50% - dimer/tetramer balance in solution.

**Table 2:** SAXS structural parameters for the experimental data (Native and Bound protein) and for the simulated SAXS profile of the crystallographic structures (Monomer, Dimer and Tetramer).

Parameters	Native	Monomer	Mon/Dim fit	Bound	Dimer	Tetramer
R_g_ (Ǻ) (Guinier)	16.1	—	15.8	23.8	—	—
R_g_ (Ǻ)	15.9	14.2	—	24.7	21.6	26.3
D_max_ (Ǻ)	50.0	49.5	—	79.0	78.5	92.3
Mol. Mass (kDa)	9.5	13.7	—	38.8	27.4	54.8

### Crystal structure of the MjTX-I/suramin complex and its comparison with native MjTX-I

**Figure 4:**
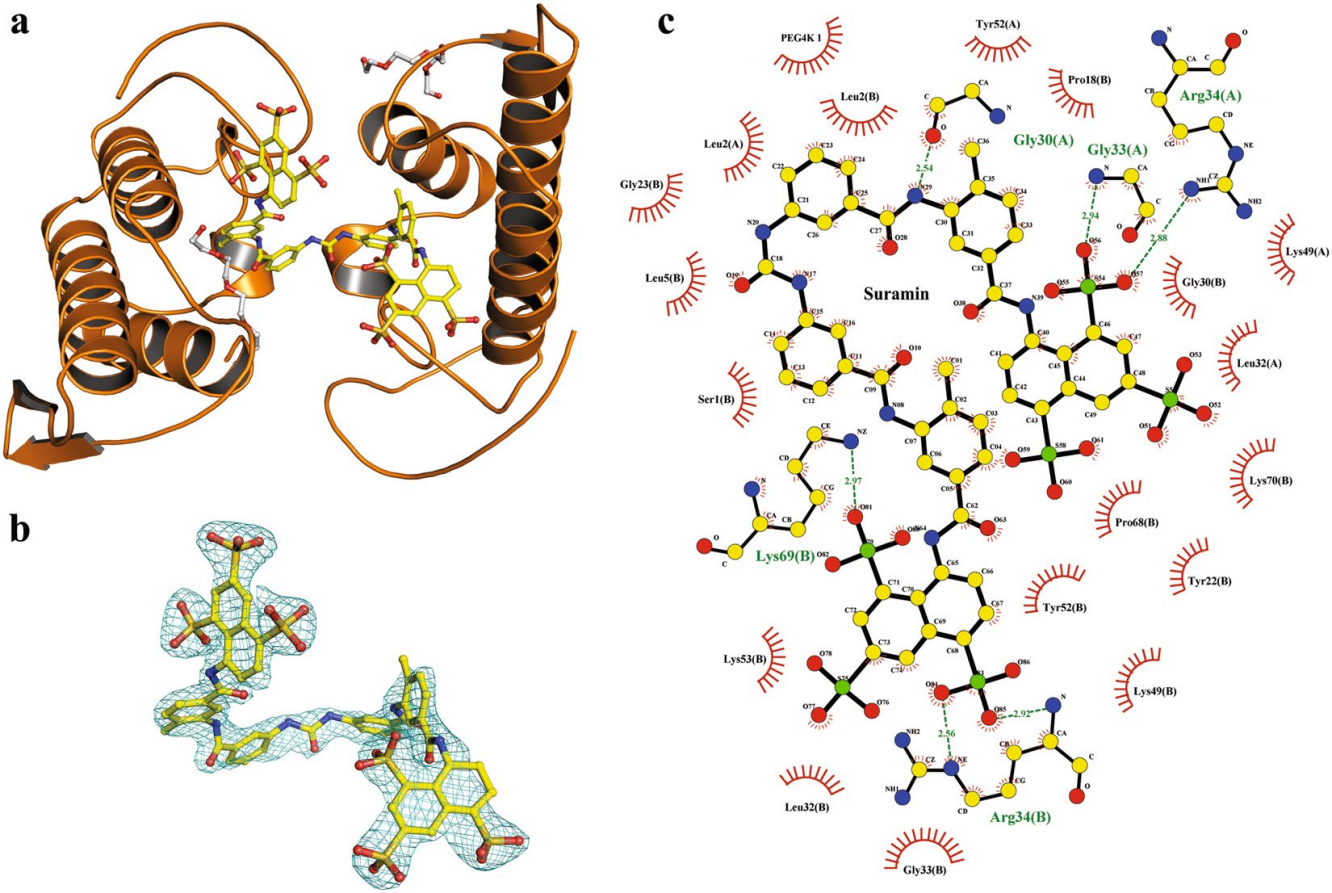
Crystal structure of the MjTX-I/suramin complex. (**a**) The overall structure of the MjTX-I/suramin complex is shown as a cartoon representation. Suramin molecule (yellow) are illustrate as stick representation. (**b**) Omit electron density map (coefficients 2|F_obs_| − |F_calc_|) corresponding to the suramin molecule is contoured at 1.2σ. (**c**) Interaction of suramin molecule in the MjTX-I structure. The representation of the interactions of suramin was depicted as polar contacts (broken lines) and hydrophobic contacts (arcs with radiating spokes).

The crystal structure of the complex MjTX-I/suramin was solved at 2.14 Å resolution and revealed an asymmetric unit (AU) composed of two protomers (identified as A and B) arranged according to the “compact dimeric” assembly^38^ in the P2_1_2_1_2_1_ space group (Table 3, Fig. 4a). The final refinement converged to a R_work_ value of 22.1% (R_free_ = 24.9%), and the final model was composed of 161 solvent molecules, two PEG 4000 molecules (one located close to His48 of the monomer A and the other close to Lys7 of monomer B), and one suramin molecule (located at the hydrophobic channel, interacting with residues of both monomers) (Fig. 4c).

**Table 3:** X-ray data collection and refinement statistics.

Unit cell (Å)	a = 48.7; b = 60.3; c = 102.3
Space group	P2_1_2_1_2_1_
Resolution range (Å)	51.9–2.1 (2.21–2.14)^a^
Unique reflections	16822 (1614)^a^
Redundancy	4.6 (4.8)^a^
Completeness (%)	98.1 (95.44)^a^
Mean I/σ(I)	9.6 (3.8)^a^
Wilson *B*-factor (Å^2^)	28.5
Molecules in ASU	2
Matthews coefficient V_M_ (Å^3^Da^−1^)	2.73
*R*_*sym*_ (%)	12.6 (36.4)^a^
Reflections used in refinement	16817 (1613)^a^
Reflections used for *R*_free_	850 (92)^a^
*R*_*work*_ (%)	22.1 (20.8)^a^
*R*_*free*_ (%)	24.9 (30.1)^a^
No. of non-hydrogen atoms	
Protein	1882
Waters	181
Suramin molecules	1
PEG molecules	2
CC (suramin)	0.924
Average *B*-factor	
Overall	30.6
Macromolecules	28.7
Ligands	48.7
Solvent	40.1
Ramachandran plot (%)	
Favored	95.4
Outliers	0.42
Rotamer outliers (%)	0.97
C_β_ outliers	0
Clashscore	8.25
RMS (bonds) (Å)	0.004
RMS (angles) (°)	0.90
RMS (*B*-factors for bounded atoms) (Å^2^)	13.8

^a^Numbers in parenthesis are for the highest resolution shell.

**Figure 5:**
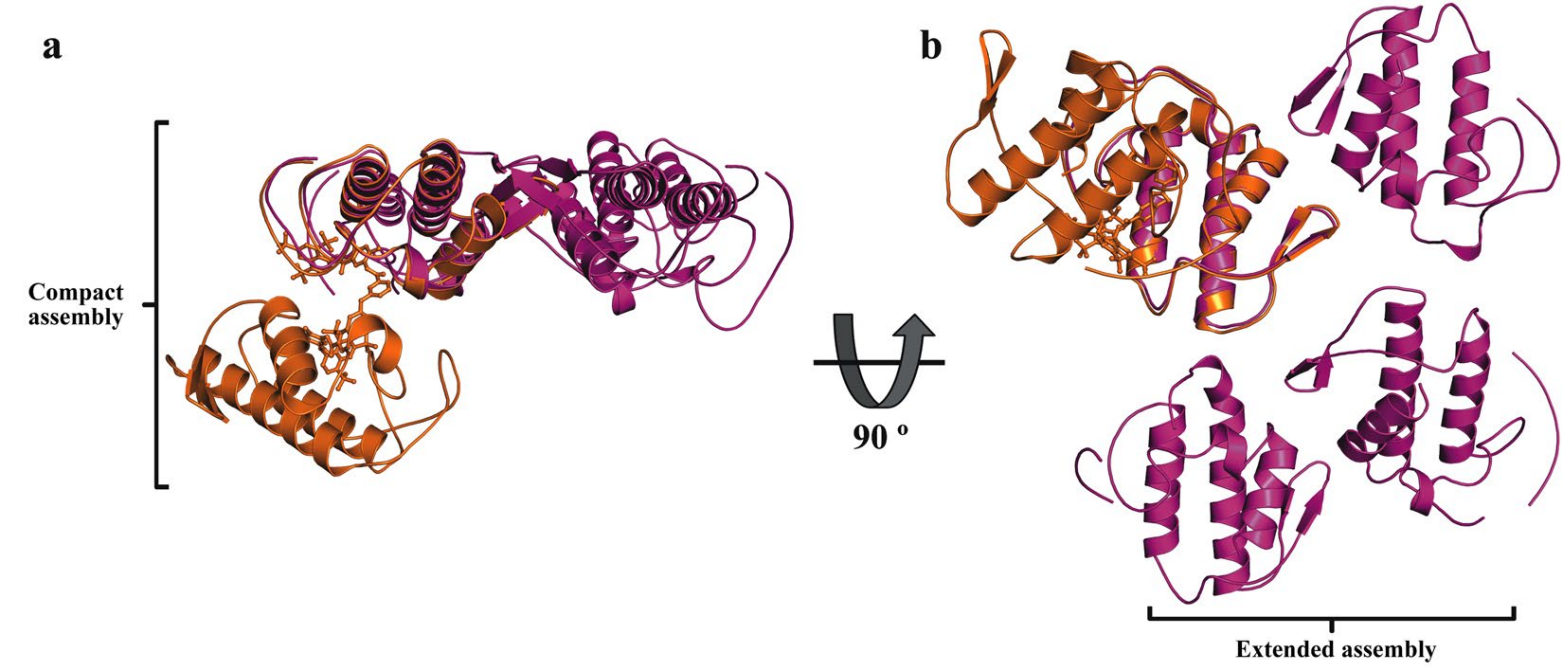
Superposition of native MjTX-I (magenta) and MjTX-I/suramin (orange) crystal structures shown as a cartoon representation. (**a**) The dimeric “compact assembly” of the MjTX-I/suramin complex is highlighted. (**b**) Two dimeric “extended assembly” forming a tetrameric arrangement of the native MjTX-I is highlighted.

Comparison of the monomers of native MjTX-I and the monomers of the toxin complexed with suramin yielded an average RMSD lower than 0.8 Å, revealing that ligand binding did not affect the tertiary structure of the protein. However, its oligomeric structure is totally different (Fig. 5). While the native MjTX-I crystal structure is composed of two dimers in an “extended assembly” that gives rise to a tetrameric array, the MjTX-I/suramin complex presents a dimeric “compact assembly”. Notably, the tetrameric arrangement seen with the MjTX-I/suramin complex may be a crystallization artefact due to the crystallization buffer that possessed high ionic strength buffer. This notion is akin to the observation made in SAXS experiments reported previously^38^, where a predominantly dimeric species similar to the “extended assembly” in the crystal structure was observed when using a low ionic strength buffer; in contrast, a monomeric species was observed when using water as solvent. The structural differences between native and ligand-bound MjTX-I suggest that the binding of suramin to the toxin causes the formation of a dimer as a “compact assembly”.

### Assessment of quaternary structure *in silico*

**Figure 6:**
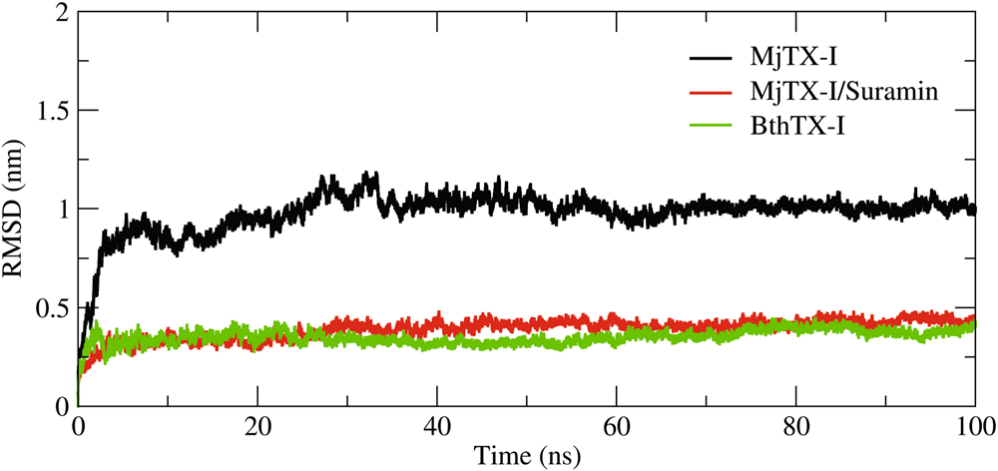
Average backbone r.m.s.d. during 100 ns molecular-dynamic simulations of the dimeric assembly of BthTX-I (green), MjTX-I with (red) and without (black) suramin ligand. The simulations demonstrated that the bound assembly (red) and the BthTX-I (green) presents a lower average r.m.s.d. value and a lower level of r.m.s.d. fluctuations compared to the unbound assembly (black).

Molecular dynamic simulations were performed to check the stability of the dimeric conformation model based on the crystal structure obtained in this work: native MjTX-I (dimeric assembly without ligands - “compact assembly”) and the complex MjTX-I/suramin (dimeric assembly bound to suramin). RMSD calculations for each system presented a very different fluctuation pattern (Fig. 6). Native MjTX-I (compact assembly) presented a high RMSD - approximately 10 Å - changing its starting conformation in the first nanoseconds of simulation and acquiring an oligomeric conformation that was distinct from the initial crystallographic dimer. On the other hand, the MjTX-I/suramin complex presented a very stable RMDS fluctuation (always less than 5 Å) and maintained the quaternary conformation along the simulations.

The presence of suramin was able to stabilize the “compact dimer” of MjTX-I, inducing the toxin to a RMSD fluctuation that was very similar to those found for BthTX-I (PLA_2_-like toxin isolated from *B. jararacussu*) when simulated in similar conditions (Fig. 6). The presence of tyrosine in position 119, which is found in most PLA_2_-like proteins, may play a role in dimer assembly by interactions of Tyr119 and Lys20 side chain residues, as observed in BthTX-I simulations. However, in MjTX-I, these interactions are missing due the Tyr119Val substitution, which explain its monomeric conformation or its dimeric conformation in the “extended dimer” arrangement for particular physical-chemical conditions, as previously shown^34^. Therefore, the compact dimeric assembly can be only reached when MjTX-I is in the presence of suramin, since this ligand is responsible for maintenance of the dimer.

## Discussion

### MjTX-I paralyzing activity is neutralized by suramin

Despite PLA_2_-like proteins being non-neurotoxic myotoxins, *in vitro* myographic assays in rodent neuromuscular preparations have been shown to be very sensitive experimental model for investigation of the basis of their membrane destabilizing activity^39^. This occurs because the first consequence of membrane destabilization is the collapse of the ionic gradient, which leads to cell depolarization, unexcitability and muscle paralysis^4,39^. Therefore, although morphological studies have clearly demonstrated that PLA_2_-like proteins can induce muscle damage, functional myographic approaches revealed the early stages of this toxic effect^40^.

Functionally, a myotoxic effect is characterized by the blockade of both indirect twitches (evoked via nerve) and direct twitches (elicited by the muscle stimulation), in contrast to a neurotoxic effect that leads to the exclusive blockade of indirect twitches. In this way, we have previously characterized the myotoxic effect of MjTX-I in a mouse phrenic-diaphragm preparation^34^. We showed that although it was a weaker myotoxin when compared to other PLA_2_-like toxins in the experimental conditions (5 µM)^17,41,42^, MjTX-I simultaneously blockaded both direct and indirect twitches. In the present work, in order to evaluate the influence of suramin upon the myotoxic effect of MjTX-I by means of myographic study, it was necessary to avoid elevated concentrations of drugs in the bath medium, which could affect the function of the neuromuscular preparation. Thus, we observed that, at a lower concentration (2.5 µM), MjTX-I is still able to blockade the indirect contractions with a t_1/2_ value (32.5 ± 5.6 minutes; n = 4), data that are not significantly different from those previously found to blockade both direct (39.5 ± 5.3 minutes; n = 4) and indirect (29.6 ± 1.7 minutes; n = 3) contractions^34^. Therefore, considering these findings and the fact that the recording of indirect contractions does not require the presence of a neuromuscular blockade in the bath medium, a condition that is essential for the recording of a direct contraction, this work was based only on the recording of indirect contractions.

The present study showed that pre-incubation with suramin prevents typical paralysis induced by MjTX-I in a phrenic nerve-diaphragm muscle preparation, probably due to an inhibition of its myotoxic effect. This result is consistent with previous works that showed the ability of suramin to inhibit the neuromuscular blockade induced by PLA_2_s-like myotoxins^17,22^.

### Is the particular oligomeric conformation of MjTX-I related to its lower myotoxic activity

Previous studies^34^ have demonstrated that MjTX-I is a protein that can present different oligomeric states in solution. It exhibits a monomeric form when in ultra-pure water or low ionic strength buffer, but higher oligomeric states are observed in higher ionic strength buffered solutions^34^. After protein purification, its behaviour in solution was investigated with DLS and SAXS experiments, either using MjTX-I in its native form and with the protein in the presence of suramin (Fig. 3). DLS assays demonstrated MjTX-I oligomerizes when suramin is added to the protein solution, since the toxin hydrophobic radius (R_H_) increases from 2.0 to 3.8 nm. Indeed, SAXS assays also demonstrated that the native MjTX-I is predominantly monomeric, but in the presence of suramin, the toxin oligomerizes. These data are in agreement with ITC assays, which display a biphasic shape in thermograms, suggesting the occurrence of two phenomena: *(i)* suramin binding to the toxin, and *(ii)* protein dimerization. A similar thermogram was observed with ITC assays for the MjTX-II/suramin complex^22,43^, in which suramin binding to the toxin led to protein oligomerization (tetrameric conformation in this case). Thus, DLS, SAXS and ITC experiments suggested that MjTX-I dimer formation is a consequence of suramin binding. Furthermore, despite the particularities of suramin binding sites for different toxins, the suramin-induced oligomerization tendency was also observed for Ecarpholin S/suramin, and for MjTX-II/suramin^22,43^.

Molecular dynamic simulations with MjTX-I in the “compact dimer” assembly with and without suramin also demonstrated that only the complex is stable. This result is in contrast to the assays performed with a typical PLA_2_-like toxin (BthTX-I), in which its native dimeric structure (“compact dimer”) is stable along with simulation. These data reinforced that the “compact dimer” conformation of MjTX-I is only stabilized by binding of the suramin inhibitor at its interface. However, as demonstrated by us previously^34^, the unbound structure (native MjTX-I) may eventually present higher oligomeric conformations (dimeric or tetrameric), but in this case, they are formed by the “extended dimer” assembly.

It has been observed in previous works with different PLA_2_s-like toxins^30^ that the interchain Tyr119 interaction may play a role in stabilization of the compact dimer for the toxin-membrane interaction. According to these studies, when the toxins are in their active state, they are able to dock and disrupt membranes using two different protein regions (MDoS and MDiS regions), and the interchain Tyr119 interaction is present in all cases in this state, except for MjTX-I (present work) and MjTX-II (this toxin presents an interchain Tyr119/Asn17 interaction^44^). Interestingly, MjTX-I presents important natural substitutions compared to other PLA_2_-like toxins, particularly Arg118Asp (a residue that comprises the MDoS) and Tyr119Val, which plays an important role in dimeric assembly. Furthermore, four other exclusive natural substitutions were found in the C-terminal of MjTX-I, the largest among all bothropic PLA_2_-like toxins^34^.

**Figure 7:**
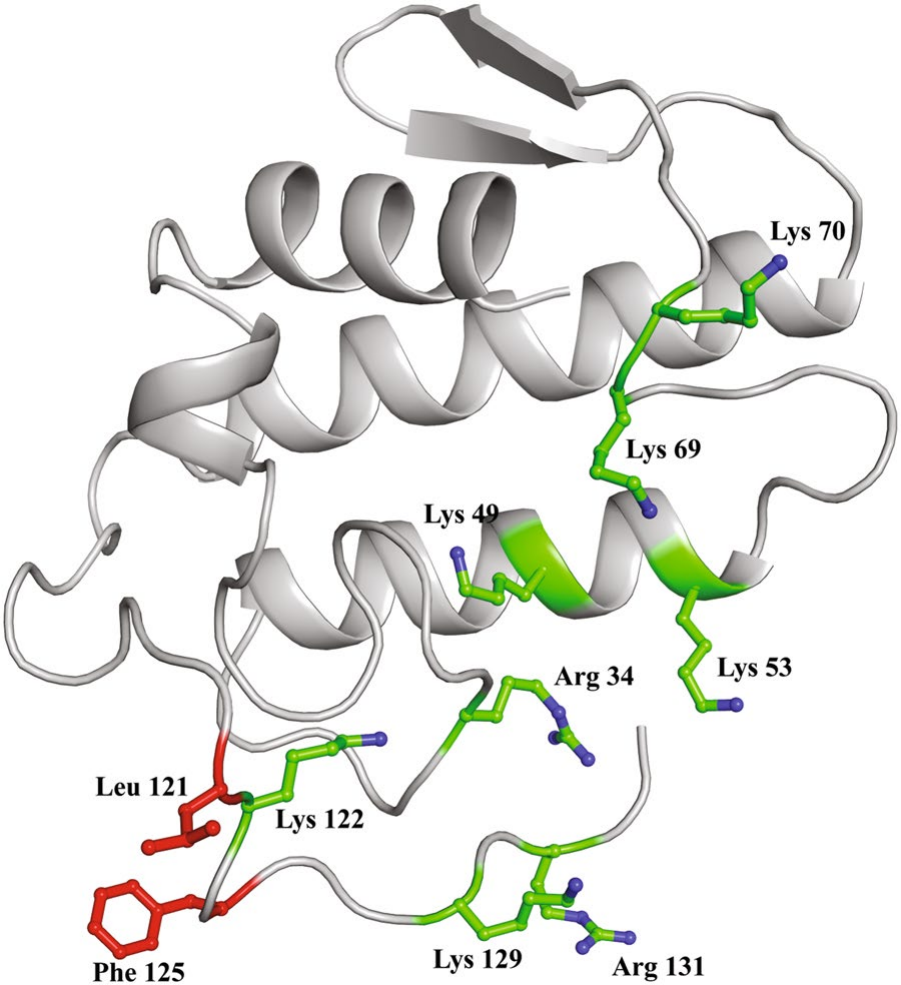
Functional sites in monomeric structure of MjTX-I. The putative-MDoS composed by positive residues are represented as green sticks and the MDiS composed by hydrophobic residues is represented with red sticks.

The particular MjTX-I structural conformation and its sequential natural substitutions may explain the lower myotoxic activity observed in functional experiments compared to other PLA_2_-like toxins^34^. Evidences obtained by structural studies (in solution and crystallography) led us to hypothesize that MjTX-I myotoxicity is expressed with the toxin in the monomeric state. In this conformation, the dynamic myotoxic mechanism recently proposed for PLA_2_-like toxins^38^ is not present, weakening the disruption mechanism. Furthermore, the MDoS for MjTX-I presents just two basic residues, in contrast with six basic residues for dimeric PLA_2_-like toxins, which weaken the docking process. However, a deeper analysis of the MjTX-I monomer structure revealed an interesting feature: a basic cluster consisting of 5–7 Lys/Arg residues is located in a protein face exposed to the solvent (Fig. 7). Several of these residues are conserved in PLA_2_-like toxins (Sup. Fig. 1), and sulfate ions are often found bound to this region in PLA_2_-like crystal structures. As previously shown, PLA_2_-like toxins have their potency decreased when in a monomer assembly^45^, but they are still active. Thus, we suggest that this region could be an alternative MDoS region for monomeric PLA_2_-like toxins (described here as a putative-MDoS). Indeed, as will be discussed in the next section, the suramin inhibitor binds in this region. Furthermore, the MDiS region is also on this face of the toxin, which allows the proposed myotoxic mechanism to remain possible.

### MjTX-I inhibition process by suramin

**Figure 8:**
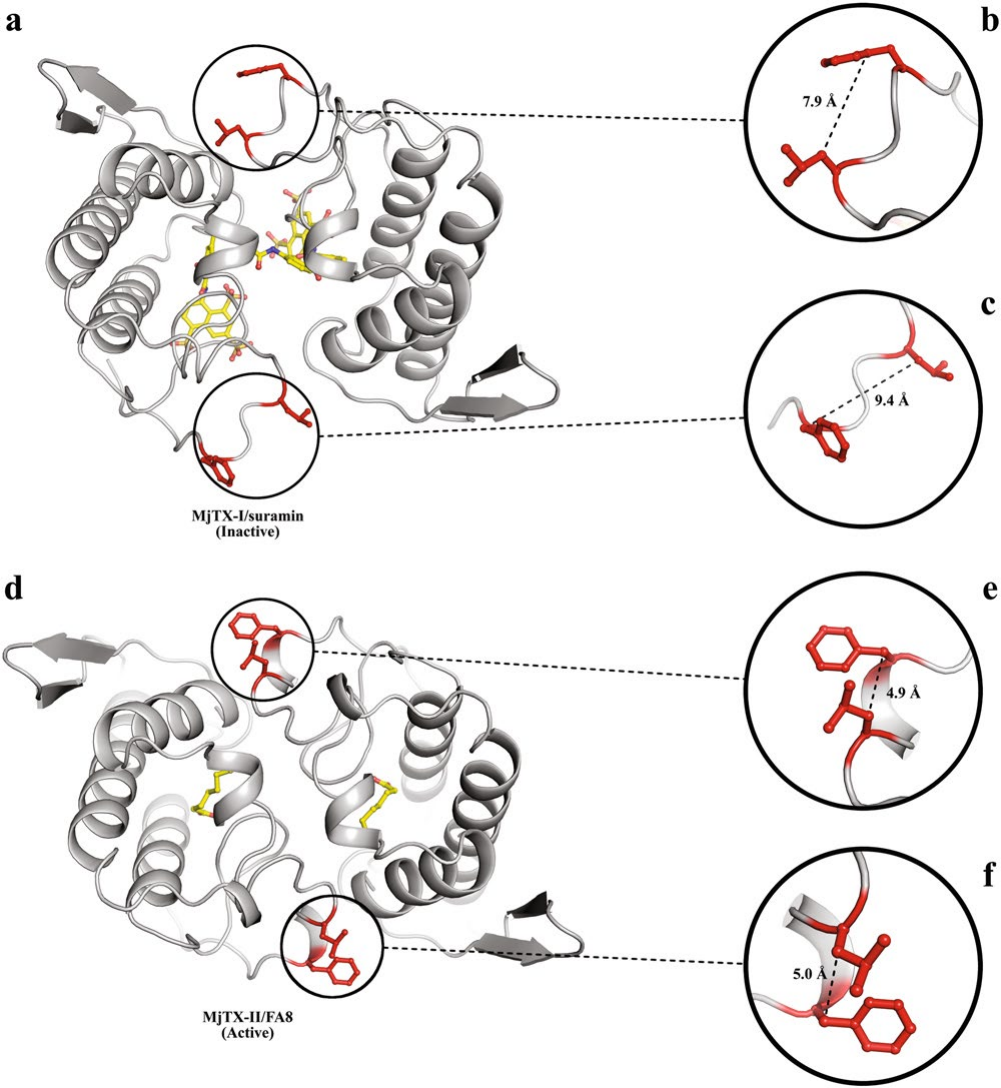
Differences of distances from functional MDiS (red sticks) from MjTX-I/suramin and MjTX-II/FA8. MDiS distance is obtained measuring the distance between Leu121 Cβ and Phe125 Cβ. (**a**) Crystal structure from MjTX-I/suramin (inactive). (**b**) Zoomed MDiS from monomer A (7.9 Å). (**c**) Zoomed MDiS from monomer B (9.4 Å). (**d**) Dimeric structure ofMjTX-II/FA8 (active). (**e**) Zoomed MDiS from monomer A (4.9 Å). (**f**) Zoomed MDiS from monomer B (5.0 Å).

The MjTX-I/suramin crystal structure displays two toxin protomers that interact through one suramin molecule, leading to a PLA_2_-like compact dimeric assembly formation^15,30^. Thus, at first sight, the MjTX-I/suramin complex dimerizes, and its MDoS and MDiS regions become aligned in a similar manner to other dimeric PLA_2_-like toxins when they are in their “active state”^22,31,33,46^. However, a deeper analysis showed that there are important distortions in the residue configurations that compose the MDiS region. The distances between C_β_ from the residues (Leu122 and Phe125) were between 8–9 Å for both monomers (Fig. 8a–c). According to previous studies^46^, these residue arrangements are found in one of two monomers of PLA_2_-like toxins when they are in an inactive conformation, and this distorted monomer was called a non-canonical monomer. Thus, according to this classification, both monomers of MjTX-I/suramin are in the non-canonical conformation and are thus unable to interact with membranes. By contrast, PLA_2_-like toxins in the active conformation (both monomers in the canonical conformation) present distances between C_β_ from the residues (Leu122 and Phe125) of approximately 5 Å for both monomers (Fig. 8d–f)^46^.

**Figure 9:**
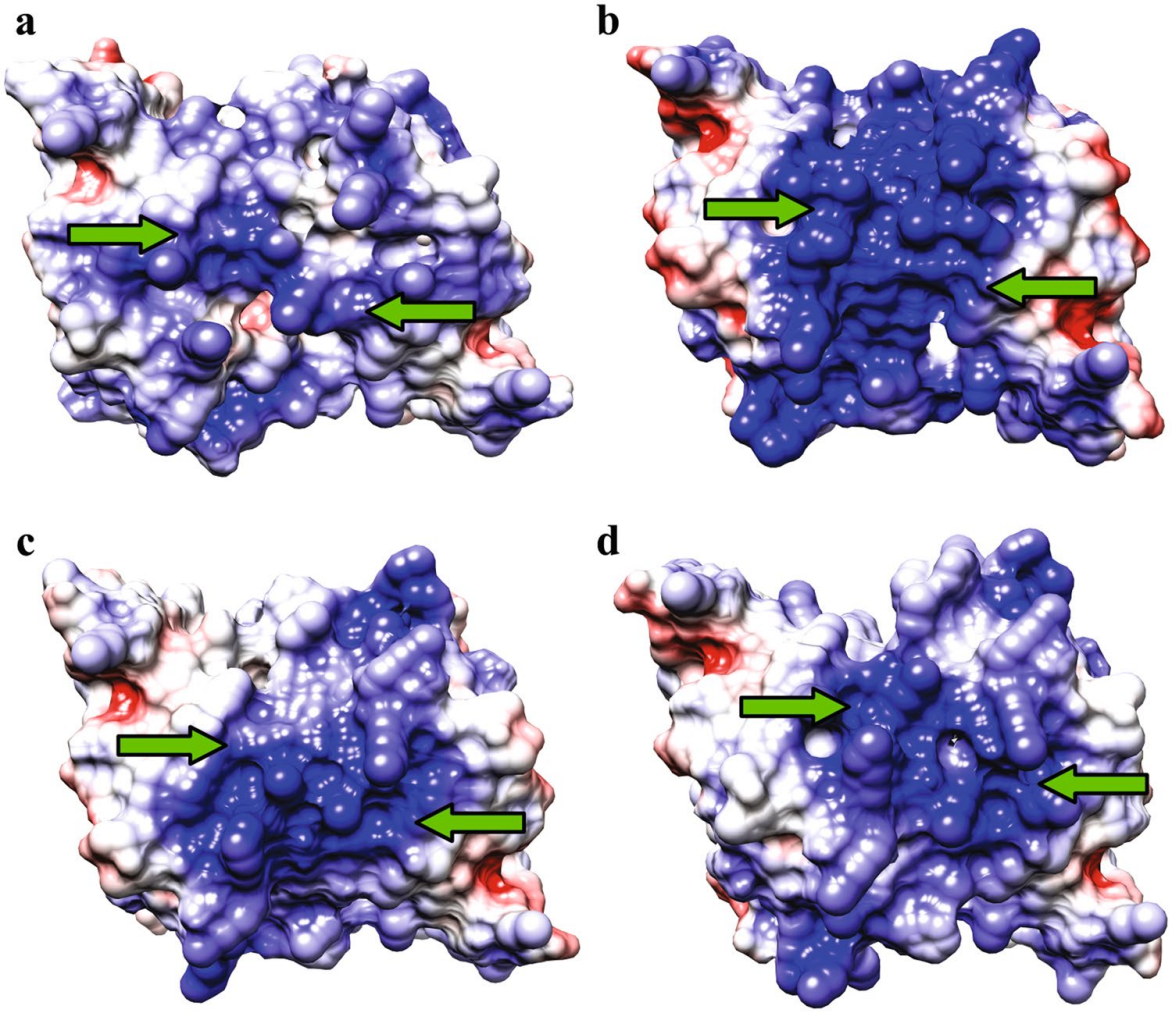
Electrostatic surface charged distribution of dimeric PLA_2_-like structures in active state and MjTX-I/suramin. All structures are in the same orientation and the green arrows are pointing the MDoS. (**a**) MjTX-I/suramin complex, (**b**) MjTX-II (PDB id - 4KF3), (**c**) BthTX-I/PEG4K (PDB id - 3IQ3) and (**d**) MTX-II (PDB id - 4K06).

Another interesting feature observed in the MjTX-I/suramin structure is that, in contrast to other PLA_2_-like toxins^22,31,33,46^, its surface displays few basic residues exposed to the solvent (Fig. 9). Two reasons may be listed. The first one is related to the natural substitution Arg118Asp from the MDoS region of the MjTX-I. The second is related to the binding of the suramin molecule that hides several basic residues, particularly Arg34, Lys49, Lys53, Lys69, and Lys70 (Fig. 7), which are related to the putative-MDoS suggested in the previous section. Similarly, Murakami *et al*.^15^ also observed important surface electrostatic charge differences due to suramin binding to BaspTX-II, and this fact was attributed by the authors as the main cause of toxin inhibition^15^.

Thus, MjTX-I inhibition by the suramin may be related to two causes: *(i)* distortion of MDiS from both monomers impairing the membrane disruption mechanism by the toxin; and *(ii)* surface electrostatic changes of the complex that interfere with the toxin membrane dockage process (putative-MDoS is partially hidden).

### PLA_2_-like inhibition mechanism by suramin

Suramin has been tested as an inhibitory molecule for different PLA_2_-like toxins, including BthTX-I from *B. jararacussu*^17^, myotoxin-II from *B. asper*^15^, MjTX-II from *B. moojeni*^22^ and MjTX-I (present work). Crystal structures of PLA_2_-like toxins complexed to suramin have also been described for four different PLA_2_-like toxins: myotoxin-II^15^, MjTX-II^22^, MjTX-I and Ecarpholin S^43^) in order to understand the structural bases of this inhibitory mechanism.

**Figure 10:**
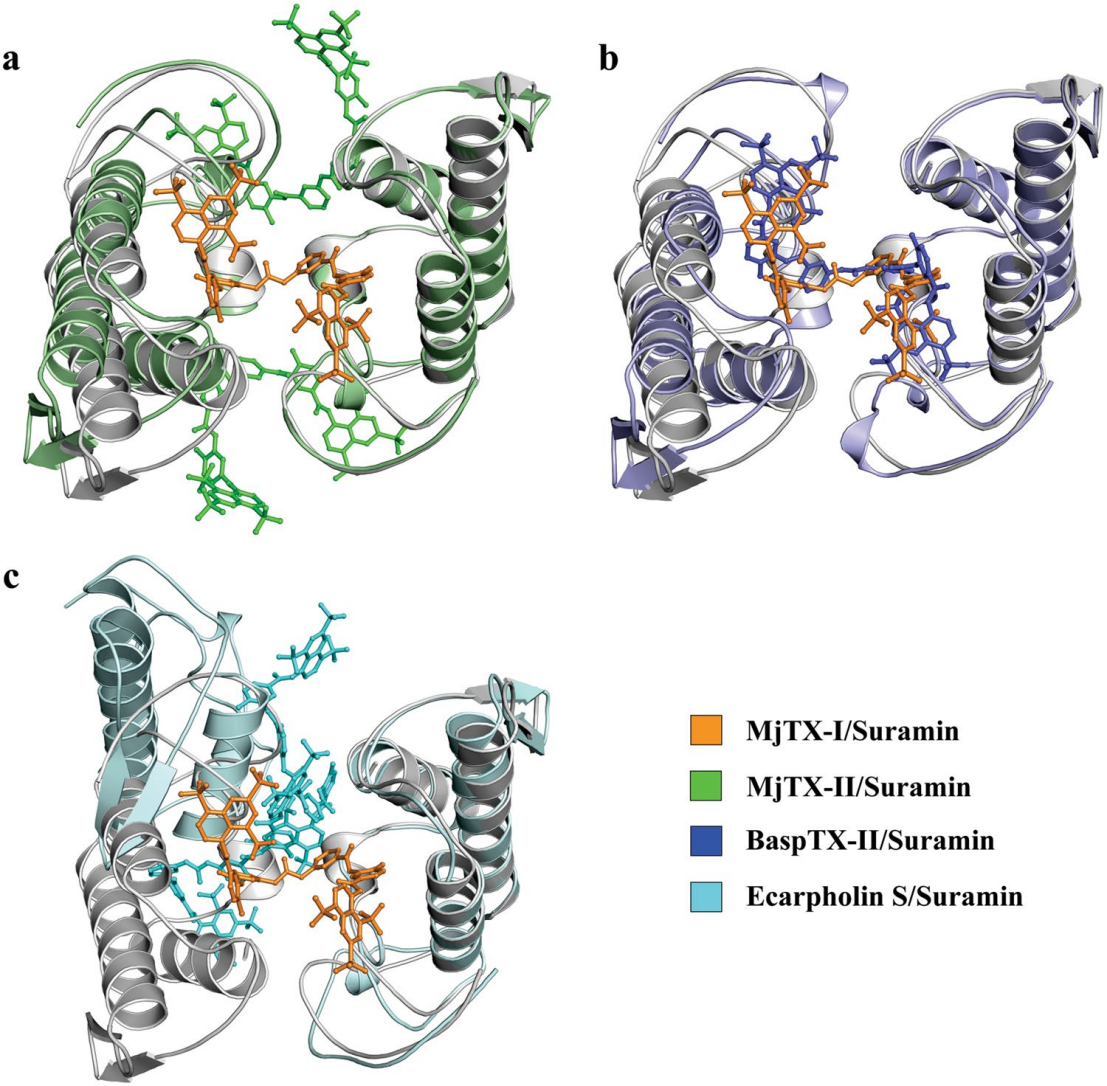
Superposition of the MjTX-I/suramin crystal structure complex and other PLA_2_s-like complexed to suramin shown as a cartoon and sticks representations. (**a**) MjTX-I/suramin (protein in gray and suramin in orange) and MjTX-II/suramin (green). (**b**) MjTX-I/suramin (protein in gray and suramin in orange) and BaspTX-II/suramin (blue). (**c**) MjTX-I (protein in gray and suramin in orange) and Ecarpholin S/suramin (cyan).

However, the crystal structures presented remarkable differences in their oligomeric structures and on the ligand binding sites (Fig. 10). A likely reason may be the rather negative electrostatic surface potential of the ligand as well as its conformational flexibility, which allows binding to different sites of different proteins. Interestingly, despite the high sequential and structural similarity of PLA_2_-like toxins, few natural mutations in these proteins may lead to different binding sites, which may also lead to oligomeric changes induced by this ligand.

Despite the diversity of the ligand-protein interactions, the oligomerization process seems to be a common feature in most PLA_2_-like/suramin complexes. The dimeric structure of Ecarpholin S^43^ presents an octameric structure when complexed to suramin; dimeric MjTX-II^22^ oligomerizes to a tetramer in the presence of this ligand and the monomeric MjTX-I forms a dimeric arrangement in the presence of the ligand (present work). BaspTX-II is the unique exception, in which both native and complexed structures present a dimeric conformation despite the oligomeric changes observed in this structure^38^.

The inhibitor interactions with the toxins are also distinct in these structures: the MjTX-II/suramin is complexed to two suramin molecules, which interact on the external area of each toxin monomer, being one of the sulfonated naphthyl rings close to MDoS; the other is close to the MDiS^22^ (Fig. 10a). Suramin binds to Ecarpholin S^43^ simultaneously to the C-terminal, N-terminal and interfacial recognition face (*i*-face) (Fig. 10c). In contrast, BaspTX-II/suramin and MjTX-I/suramin structures present only one inhibitor molecule, which is located in the hydrophobic channel of each considered complex. Suramin molecules establish similar interaction with both proteins, including the strictly conserved residues for PLA_2_-like toxins: Gly30, His48, Tyr52 and Lys69 and other basic residues Arg34, Lys49, Lys53 and Lys70 (Fig. 10b, Sup. Fig. 2). The authors of the BaspTX-II/suramin study suggested that suramin, while interacting at this position, shifts the *i*-face of BaspTX-II from a positive charge region to a negatively charged region, making the protein unable to bind on the membrane. Taking into account that the suramin binds in the same regions of MjTX-I and BaspTX-II, similar methods for inhibiting the myotoxic activity for both proteins should occur.

Thus, all these structural studies demonstrated that suramin inhibits different PLA_2_-like toxins by blocking their active sites (MDoS and/or MDiS), and despite the differences in the binding regions and eventual oligomeric changes suffered by the protein, this molecule is an efficient inhibitor against myotoxic effects caused by snake bites.

## Concluding Remarks

*B. moojeni* snake venom seems to present interesting particularities regarding PLA_2_s-like myotoxicity expression. As has been demonstrated by us in the present and previous studies, MjTX-I^34^ and MjTX-II^22,44^ have peculiar myotoxic mechanisms due to differences found in their amino acid sequences that are reflected in their structures and ligand binding. Notably for MjTX-I, these structural differences lead to important reduction in its myotoxic activity. Thus, these facts need to be taken into account for the development of specific neutralizing agents against this venom. However, despite the different inhibition mechanisms by suramin against different PLA_2_s-like toxins, this ligand seems to be an efficient neutralizing agent for all tested PLA_2_s-like toxins and needs to be considered either as a potential inhibitor or as the molecular basis for new inhibitors search of local myotoxic effects caused by different ophidian accidents.

## Experimental Procedures

### Toxin isolation and suramin source

Crude venom (freeze-dried) was diluted in 50 mM ammonium bicarbonate (pH 8.0) and submitted to ion exchange chromatography. Fractions corresponding to MjTX-I were obtained by a gradient of 50 mM to 500 mM ammonium bicarbonate (pH 8.0), as previously described^47^. This fraction was submitted to a gradient of 0–66.5% acetonitrile in 0.1% trifluoroacetic acid in reverse phase chromatography to improve the sample purity. Suramin sodium salt (S2671) was obtained from Sigma Chemical Co. (St. Louis, MO, USA).

### Functional studies

Male Swiss mice (25–30 g) were sacrificed by exsanguination after cervical dislocation. The phrenic nerve-diaphragm muscle preparations were removed and mounted vertically in a conventional organ-bath containing 15 mL of physiological solution (mM): NaCl, 135; KCl, 5; MgCl_2_, 2; NaHCO_3_, 15; Na_2_HPO_4_, 1; glucose, 11. This solution was continuously gassed with 95% O_2_ and 5% CO_2_ and maintained at 35 ± 1 °C. The preparation was attached to an isometric force transducer (Grass, FT03) coupled to a signal amplifier (Gould, 13–6615–50). The recordings were made on a computer through a data acquisition system (*Gould Systems, Summit ACQuire* and *Summit DataViewer*). Indirect contractions were evoked by supramaximal pulses (0.2 Hz, 0.5 ms) delivered from an electronic stimulator (Grass, S88K) and applied to the phrenic nerve by means of a suction electrode. Preparations were allowed to stabilize for 45 minutes before the addition of MjTX-I (2.5 µM), suramin (125 µM) or a mixture of MjTX-I plus suramin pre-incubated at 35 °C for 15 minutes. After administration, the amplitudes of indirect twitches were measured for 90 minutes. The results of the myographic study were expressed as the mean ± S.E. and were analysed with Student’s t-test. Values of p < 0.05 were considered significant.

Institutional Animal Care and Use Committee (Institute of Biosciences - Sao Paulo State University - UNESP) approved this study under the number 033/05. Animal procedures were in accordance with the guidelines for animal care prepared by the Committee on Care and Use of Laboratory Animal Resources, National Research Council, USA.

### Isothermal titration calorimetry

Calorimetric experiments were performed in a microcalorimeter iTC200 (GE Healthcare). Interactions between the macromolecules were determined at 25 °C by the injection of suramin (750 μM) into the reaction cell containing MjTX-I (50 μM) through 20 injections of 2 μL with a spacing interval of 240 s. Both samples (MjTX-I and suramin) were diluted in ammonium bicarbonate buffer (50 mM, pH 8.0), and the heatsof dilution and mixing of suramin were determined in control experiments and subtracted from the titrations. All titrations were performed in triplicate.

The thermogram areas of peaks were determined automatically with *Origin v.7.0 Add-on* provided by the equipment manufacturer, and curve fitting was performed by binding polynomials as previously described^22,35^.

### Dynamic light scattering

The dynamic light scattering (DLS) experiments were executed at 291 K using a DynaPro TITAN ^TM^ device (Wyatt Technology^TM^) with native MjTX-I and MjTX-I/suramin at 2.5 mg.mL^−1^ (MjTX-I:suramin 1:10 respectively). Measurements were carried out with the protein in 20 mM ammonium bicarbonate (pH 8.0). One hundred measurements were acquired in each experiment. Analysis of the final data was performed with the *Dynamics v.6.10* program (Wyatt Technology^TM^).

### Small Angle X-ray Scattering

Small angle X-ray scattering (SAXS) data were collected at the Brazilian Synchrotron Light Laboratory (LNLS – CNPEM, Campinas – SP) using the SAXS2 beamline. The wavelength of the incident beam radiation was set to λ = 1.55 Å, and the sample-detector distance was adjusted to 1007 mm, resulting in a scattering vector ranging from 0.013 Å^−1^ < q < 0.319 Å^−1^ (q = 4π sinθ/λ, where 2θ is the scattering angle). The scattering pattern was recorded in a MAR CCD detector (MAR Research). Data for MjTX-I were collected at 1 and 5 mg.mL^−1^ dissolved in 20 mM of ammonium bicarbonate (pH 8.5) in the absence and presence of suramin (MjTX-I:suramin 1:10), respectively, to evaluate changes in the protein oligomerization state due to the presence of the inhibitor. SAXS patterns were corrected for the detector responses and scaled by the incident beam intensity and sample absorption. The background scattering curve was subtracted from the corresponding sample scattering. Integration of the bi-dimensional SAXS patterns were performed using *Fit2D* software^48^.

Guinier analysis of the radius of gyration (R_g_) was performed using Primus software^37^. Fitting of the experimental data and evaluation of the pair-distance distribution function *p(r)* were conducted using *Gnom* software^49^. Molecular weight and oligomerization state evaluations were conducted using *SAXSMoW*^50^. *Crysol* software^51^ was used to simulate the scattering profile of crystallographic structures and to evaluate their structural parameters, and *Oligomer* software^37^ was used to evaluate mixtures of oligomeric states, determining the percentage of each population in solution.

### Crystallization and X-ray data collection

MjTX-I purified fraction was concentrated up to 10 mg.mL^−1^ in 20 mM ammonium bicarbonate (pH 8.5), and suramin was added to the solution in order to obtain a molar ratio of 1:10. Crystals of the complex MjTX-I/suramin were obtained with the hanging drop vapour-diffusion method^52^ at 291 K from a mixture of 1 µL of protein/suramin solution and 1 µL reservoir solution, and this was equilibrated against a reservoir containing 500 µL with the following composition: 32% (w/v) polyethylene glycol (PEG) 4000, 0.1 M TrisHCl pH 8.5 and 0.15 M magnesium chloride.

X-ray diffraction data were collected from a single MjTX-I/suramin crystal at a wavelength of 1.325 Å (at 100 K) using a synchrotron radiation source (MX2 station, LNLS – CNPEM, Campinas – SP) and a MAR CCD detector (MAR Research). The crystal was mounted in a nylon loop and flash-cooled in a steam of nitrogen at 100 K with no cryoprotectant. The crystal-to-detector distance was 80 mm, and an oscillation range of 1° was used, resulting in the collection of 121 frames. Data were processed to 2.14 Å resolution using the *HKL2000* program package^53^.

### Structure determination and refinement

The MjTX-I/suramin crystal structure was solved by the Molecular Replacement Method using the*PHASER* program^54^ from *PHENIX package v.1.8.4*^55^and the monomer A atomic coordinates of the native MjTX-I structure (PDB access code 3T0R) as the search model. The modelling process was performed using the *Coot v.0.7.1* program^56^, which was also used to add PEG 4000, solvent molecules and suramin molecules to the model. The crystallographic structure was automatically refined by *PHENIX package v.1.8.4*^55^. Due to the lack of electron densities, amino acid side chains Glu86 and Lys69 in monomer A and Lys128 in monomer B were not modelled. *PHENIX package v.1.8.4*^55^ and the *MolProbity* online program (http://molprobity.biochem.duke.edu/)^57^ were used to check the general quality of the final model. Refinement statistics and other information are shown in Table 3. The coordinates of the MjTX-I/suramin structure was deposited in the Protein Data Bank (PDB) under the identification code (PDB ID code): 6CE2.

### Comparative analysis

The crystal structures of the MjTX-I/suramin complex (this work), native MjTX-I (PDB id: 3T0R)^34^, MjTX-II/suramin (Myotoxin II isolated from *Bothrops moojeni* venom - PDB id: 4YV5)^22^, BaspTX-II/suramin (Myotoxin II isolated from *Bothrops asper* venom - PDB id: 1Y4L)^15^ and Ecarpholin S/suramin (Myotoxic Ser49-PLA_2_ isolated from *Echis carinatus* venom - PDB id: 3BJW)^43^ were compared using the *Coot v.0.7.1* program^56^. Structural figures were generated using the *PyMOL v.1.3* program^58^, *LigPlot*^+^ *v.1.4.5*^59^ and images containing electrostatic surfaces were generated using the *Chimera v.1.9* program^60^.

### Molecular-dynamics simulations

MjTX-I and MjTX-I/suramin crystallographic structures were submitted to MD simulations using *GROMACS (Groningen Machine for Chemical Simulation) v. 5.0.5*^61^ under the GROMOS 54A7 force field^62^ and the simple point charge (SPC) water model. Before proceeding to the MD simulations, the protonation states of the MjTX-I were calculated using *PROPKA3*^63^ and set to a pH value of 8.0. Each system was placed in a triclinic box with a distance of 4 Å from the farthest atomand then solvated and equilibrated with NaCl 100 mM. The steepest descent algorithm was used to minimize all systems energy below 100 kJ/mol/nm, and then restraints in MjTX-I and suramin were performed under a V-rescale weak temperature coupling thermostat^64^ at 310 K for 1 ns, followed by a 1-ns step of isobaric simulation (1 bar) under a Berendsen pressure coupling barostat^65^ using position restraints. Subsequently, 100 ns of unrestrained MD simulations were performed under a Nose-Hoover thermostat^66^ and Parrinello-Rahman barostat^67^.

The suramin topology was built using an ATB (Automated Topology Builder v. 2.2) online server^68^. The resulting topologies were carefully analysed, and all charges were fixed based on the force field parameters that were used, as previously suggested^69^.

## Electronic supplementary material

Figures S1 and S2
