# Supplementary material for: Structural and functional characterization of suramin-bound MjTX-I from Bothrops moojeni suggests a particular myotoxic mechanism
Source: Sci Rep. 2018 Jul 9;8:10317. doi: 10.1038/s41598-018-28584-7 (PMC6037679; doi:10.1038/s41598-018-28584-7)
Supplement: Supplementary file 1 — Figures S1 and S2 [file 41598_2018_28584_MOESM1_ESM.pdf]

Structural and functional characterization of suramin-bound MjTX-I from *Bothrops moojeni* suggests a particular myotoxic mechanism

Guilherme H. M. Salvador, Thiago R. Dreyer, Antoniel A. S. Gomes, Walter L. G. Cavalcante, Juliana I. dos Santos, César A. Gandin, Mário O. Neto, Márcia Gallacci, Marcos R. M. Fontes

SUPPLEMENTARY MATERIAL

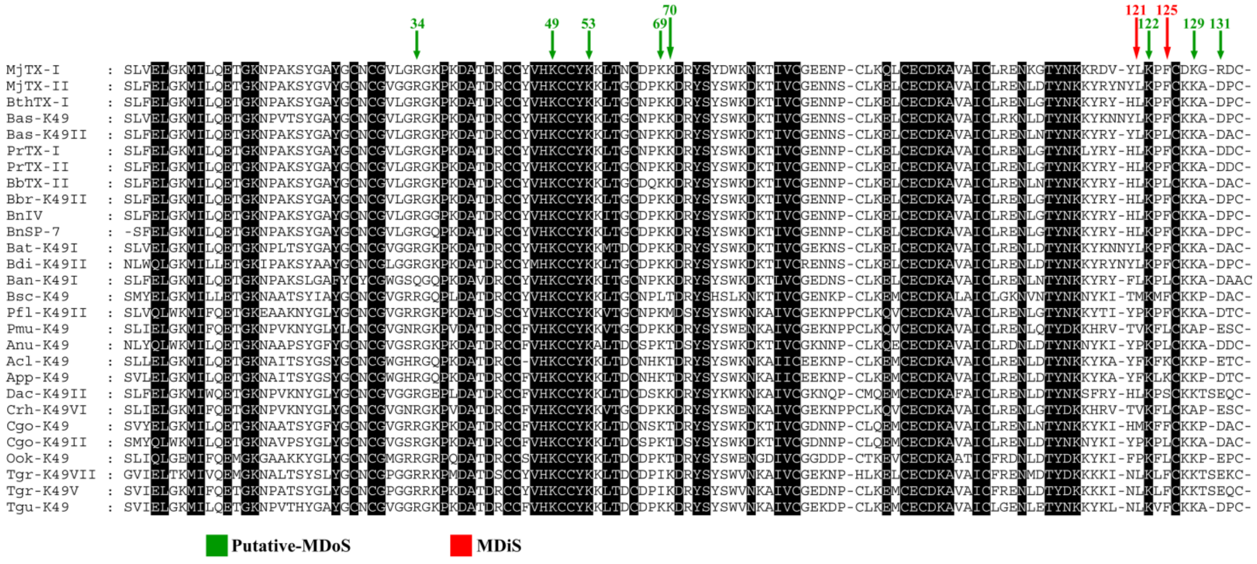

Supplementary Fig. S1. Amino acid alignment of PLA_2-like toxins from *Bothrops* genus venoms. The green arrows point to the putative-MDoS suggested for MjTX-I and the red arrows points the MDiS residues. **MjTX-I**: Myotoxin I from *Bothrops moojeni* (NCBI GI: 17368325); **MjTX-II**: Myotoxin II from *Bothrops moojeni* (NCBI GI: 62738542); **BthTX-I**: Bothropstoxin I from *Bothrops jararacussu* (NCBI GI: 51890398); **Bas-K49**: Myotoxin I from *Bothrops asper* (NCBI GI: 6492260); **Bas-K49**II: Myotoxin II from *Bothrops asper* (NCBI GI: 166215047); **PrTX-I**: Piratoxin I from *Bothrops pirajai* (NCBI GI: 17433154); **PrTX-II**: Piratoxin II from *Bothrops pirajai* (NCBI GI: 17368328); **BbTX-II**: Myotoxin II from *Bothrops brazili* (NCBI GI: 558705001); **Bbr-K49II**: MTX II from *Bothrops brazili* (NCBI GI: 557804964); **BnIV**: Lys49-PLA_2 from *Bothrops pauloensis* (NCBI GI: 333361256); **BnSP-7**: Lys49-PLA_2 from *Bothrops pauloensis* (NCBI GI: 239938675); **Bat-k49I**: Myotoxin I from *Bothrops atrox* (NCBI GI: 40888878); **Bdi-K49II**: Lys49-PLA_2 from *Bothrops diporus* (NCBI GI: 387537882).

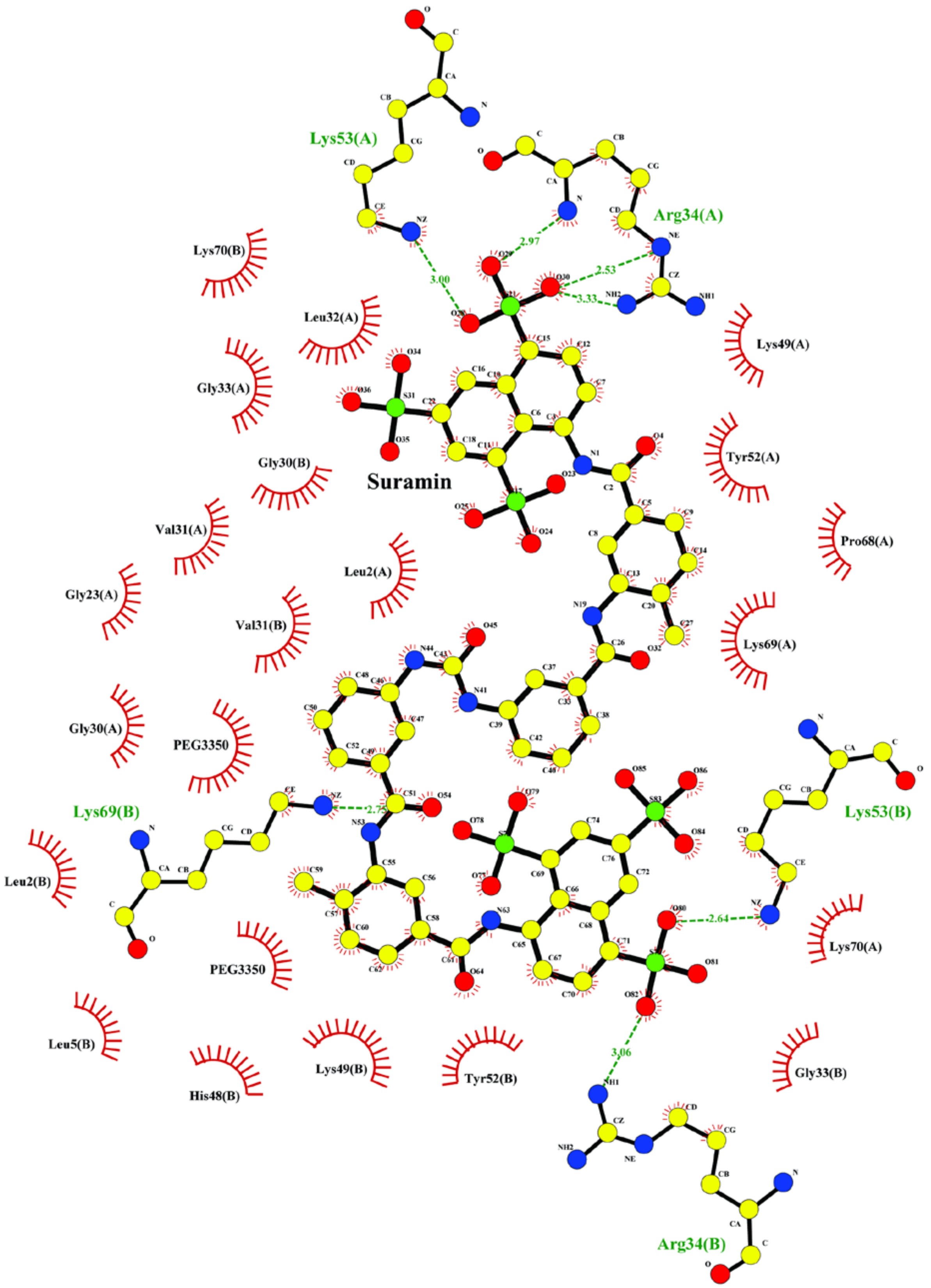

Supplementary Fig. S2 - Interaction of suramin molecule in the BaspTX-II/suramin structure. The representation of the interactions of suramin was depicted as polar contacts (broken lines) and hydrophobic contacts (arcs with radiating spokes).
